# Case report: Heart retransplant from a donor after circulatory death and extended transport period with normothermic perfusion

**DOI:** 10.3389/fcvm.2023.1212886

**Published:** 2023-08-11

**Authors:** Patpilai Kasinpila, Chawannuch Ruaengsri, Tiffany Koyano, Yasuhiro Shudo

**Affiliations:** Department of Cardiothoracic Surgery, Stanford University, Stanford, CA, United States

**Keywords:** heart retransplantation (RTx), normothermic *ex vivo* perfusion, donor distance, donor after circulatory death, organ preservation

## Abstract

A 55-year-old man with end-stage heart failure, who had an orthotopic heart transplant 21 years prior, underwent heart retransplantation using a heart from a donor with circulatory death in a distant location and an extended transport period with normothermic *ex vivo* perfusion. Owing to the persistent and worsening shortage of donor hearts, this case illustrates that expanding the donor acceptance criteria to include more distant donor locations and enrolling recipients with extended criteria (e.g., heart retransplantation) is feasible.

## Introduction

Orthotopic heart transplantation remains the gold standard of treatment for end-stage heart failure. Recently, heart transplantation from donors after circulatory death (DCD) has expanded, which was enabled by the usage of the FDA-approved transportable Organ Care System™ (OCS) (TransMedics, Andover, MA, USA) (1). This innovative device preserves the standard and extended-criteria for *ex vivo* donor hearts during normothermic *ex vivo* perfusion. Improvements in preservation and transportation conditions can improve organ quality at the time of transplantation, shorten the acceptable maximum allograft ischemic time, and optimize patient outcomes.

We describe our successful experience with a normothermic *ex vivo* perfusion system, using a heart from a DCD, for an extended transport period >7 h using our modified strategy. To our knowledge, this is the first report of heart retransplantation using a normothermic *ex vivo* perfusion system in DCDs.

## Case description

A 55-year-old man with heart failure and reduced ejection fraction (EF 30%) following a heart transplant in 2001 was listed for heart retransplantation (2). Despite maximal medical therapy, the patient's condition deteriorated, requiring an implantable cardioverter and multiple hospitalizations. The patient was considered a candidate for heart retransplantation. A 24-year-old male with a compatible blood type was identified as a suitable donor; the donor's heart had an acceptable size and sex match with the recipient (predicted heart mass, 1.36; height, 110%; body weight, 126%) and normal biventricular function. The heart was retrieved after circulatory death of the donor in a hospital located 700 miles away.

Organ donation and the subsequent withdrawal of life support were performed in the intensive care unit, which was separate from the thoracic and abdominal organ retrieval teams. Heparin was administered to the donor 5 min before withdrawal. The donor was observed after the cessation of circulation for 2 min, declared deceased, and quickly transferred to the operating room.

Median sternotomy and laparotomy were performed simultaneously with a venous cannula placed directly into the grossly distended right atrium to enable rapid collection of 1.2 L of blood to prime the *ex vivo* perfusion apparatus.

Heparin was added to the blood collection bags. An aortic cross-clamp was applied on the ascending aorta, and 1 L of cold del Nido cardioplegia solution was delivered via the aortic root. The heart was vented by cutting across the left lower pulmonary vein and the inferior vena cava at the pericardial reflection (3).

After cardioplegia was delivered, the heart was explanted with transection at the mid-aortic arch, distal to the main pulmonary artery bilaterally, across the superior vena cava at its confluence with the innominate vein.

## *Ex vivo* preservation

The donor heart was attached to the Organ Care System (OCS™) after cannulation of the aorta and pulmonary arteries. according to the manufacturer's instructions (4), the Organ Care System circuit prime was made up by mixing 1.2 L of donor blood that had been passed through a leucocyte filter (Pall LeukoGuard BC2; Pall Corporation, Port Washington, NY, USA) with 500 ml of TransMedics Priming Solution containing buffered electrolytes and mannitol. Multi-vitamins, antibiotics, albumin and steroids were added to the system. A TransMedics proprietary maintenance solution (1 L) containing isotonic electrolytes, amino acids, dextrose-insulin, and low-dose adenosine was infused at a rate of 0–30 ml/h during *ex vivo* perfusion to maintain the coronary flow within an acceptable range of 650–900 ml/min. The heart started beating spontaneously in a sinus rhythm and did not require pacing. A vent was placed via the left atrium to decompress the left ventricle. There was no PFO or atrial septal defect found in this donor. The right atrial appendage incision which was made previously for donor blood collection, superior and inferior vena cavae were securely closed. The heart was positioned such that oxygenated blood directly entered the ascending aorta, flowed down the coronary arteries, returned to the right side of the heart, and diverted into the pulmonary artery before draining into the circuit reservoir. This apparatus principally uses aortic pressure, coronary flow, and arteriovenous lactate concentrations to assess cardiac function; a lower venous concentration indicates lactate uptake and satisfactory myocardial function. An infusion of low-dose adenosine, another infusion containing adrenaline, and adjustable circuit pump flow were used to control coronary vascular resistance and heart rate to keep parameters within the following ranges: aortic pressure 65–90 mmHg, coronary flow 650–900 ml/min, and heart rate 65–100 beats per min. The coronary inflow and effluent ports on the perfusion circuit were simultaneously sampled at regular intervals to measure myocardial lactate extraction. Lactate concentrations in the perfusate were measured using an automated iSTAT analyzer (Abbott, Princeton, NJ, USA).

We began the transplantation when the perfusion and lactate profiles met the OCS parameters. Once stable OCS pump flow, several initial downward trends in serum lactate concentrations, and biventricular motion were confirmed, we administered general anesthesia and placed arterial and venous monitoring lines. The difference in arteriovenous lactate levels improved and remained stable at less than 5 mmol/L. While the heart was being transported, the recipient underwent a repeat median sternotomy. Following successful sternal reentry, extensive dissection was performed, confirming hemostasis.

As soon as the OCS arrived in the operating room, the transportable *ex vivo* perfusion was turned off, supplemented cold del Nido cardioplegia solution was delivered to the donor heart with prompt electromechanical arrest, and the heart was removed from the OCS for implantation. This reduced the total *ex vivo* heart perfusion time.

Cardiopulmonary bypass was initiated at 34°C with aortic and bicaval cannulations, and cardiectomy was performed. During this process, the heart was cooled on ice for 30 min, which was expected to reduce oxygen demand and afford adequate cellular protection (5).

## Implantation

Donor heart implantation was performed with left atrial anastomosis, followed by ascending aortic anastomosis. During heart reperfusion, the remaining cardiac anastomoses, such as the pulmonary artery, inferior vena cava, and superior vena cava, were performed using an end-to-end anastomosis technique. This modified implantation technique (6) shortened the second warm ischemic time, reduced the aortic cross-clamp time, and secured an additional reperfusion period for the implanted heart. Although no electrical activity or ventricular squeezing was found in the initial 150 min of the reperfusion period, atrioventricular conduction and normal sinus rhythm were promptly regained. At the time of separation from cardiopulmonary bypass, an inotropic agent was administered to maintain a cardiac index of 2.5 L/min/m^2^.

The total *ex vivo* heart perfusion time was 423 min. The allograft ischemic time was 107 min, including the first and second warm ischemic times of 9 min and 15 min, respectively. The recipient cardiopulmonary bypass and aortic cross-clamp times were 233 min and 50 min, respectively. The patient recovered well and was discharged on postoperative day 17. Six months after transplantation, the patient continued to have excellent graft function without any evidence of rejection.

## Discussion

This report describes a successful clinical heart retransplantation using a heart from a DCD with an extended transport period and an *ex vivo* cardiac perfusion device.

The use of organs from DCDs has been successful for heart transplantation, which has helped reduce the discrepancy between the number of patients awaiting transplantation and the number of suitable donors. Strong endorsements for such transplants by national and international regulatory bodies have led to the wider adoption of this strategy, with organs from DCDs contributing to an increasing percentage of the total number of donors worldwide. Donor selection in DCD donations is the same as donation after brain death (DBD) scenarios, avoiding size mismatch based on predicted heart mass ratio. According to OCS heart EXPAND trial, marginal donors with an anticipated total ischemic time more than 4 h or age >50 years are now included. Further cardiac evaluations, echocardiography and cardiac catheterization, are required prior to withdrawal of life support (7).

Our retrospective outcomes after 50 years of experience of heart retransplantation demonstrated inferior short-term survival compared to primary transplantation. The decision of heart retransplantation listing is made by our multidisciplinary team based on case-by-case basis and we do not reluctant to offer heart retransplantation for candidates with severe graft dysfunction and have no other options. Given the inferior outcomes, careful candidate selection is recommended to optimize donor heart utilization (2).

Following DCD transplant, the incident of severe primary graft dysfunction (PGD) is found to be higher compared to similar DBD recipients. The pathophysiology of PGD is not well addressed in DCD hearts but thought to be due to functional warm ischemia occurring during and post withdrawal of life support. The study showed DCD recipients with severe PGD required shorter duration of mechanical circulatory supports (MCS) and spent fewer days in ICU and hospital compared to similar DBD recipients suggests DCD heart procurement process may contribute to a period of delayed graft function with subsequent rapid recovery which may differ from what we observed in DBD recipients with severe PGD (8). Therefore, our institution has low threshold of using MCS postoperatively in extended-criteria donor heart recipients. We considered using peripheral veno-arterial extracorporeal membrane oxygenation (VA-ECMO) as the main strategy to support PGD patients not only it is less invasive and promptly available but also promote lower rate of mediastinal infection. Intra-aortic balloon pump (IABP) support is routinely combined with VA-ECMO for the treatment of severe PGD requiring ECMO therapy for the benefit of augmentation of coronary perfusion, provide peripheral pulsatility and promote afterload reduction. MCS will be discontinued as soon as heart function returns to normal to prevent further complications that increase with time (9).

Several cardiac perfusion devices are currently commercially available and more are being trialed. Commercially available portable *ex vivo* heart preservation systems have made it possible to maintain physiological perfusion of a donor organ coming from a distance. The device has been used for both the resuscitation and assessment of marginal hearts from DCDs for transplantation. Approximately 20% more transplantations could be done if hearts were donated after circulatory deaths (4).

**Figure 1:**
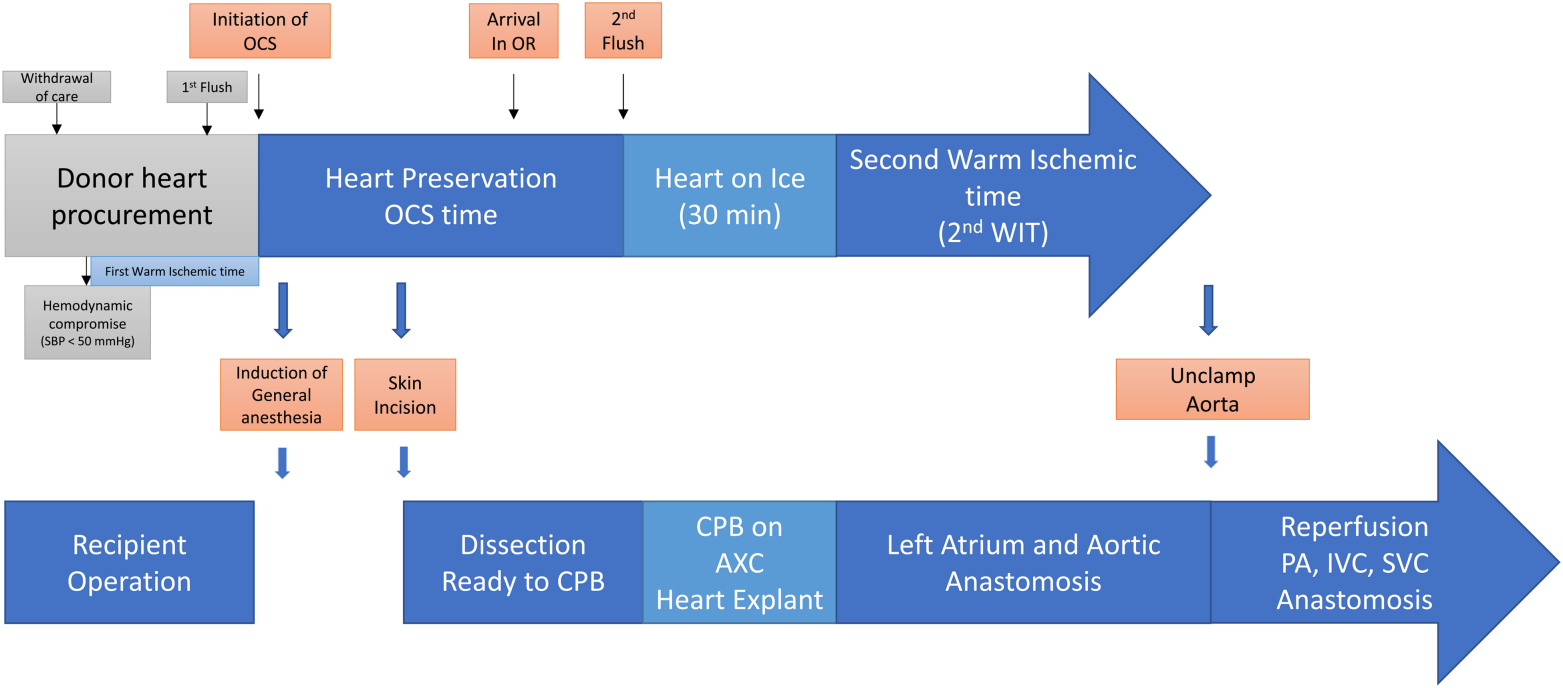
Transplanting a heart from a donor after circulatory death and extended transport period with normothermic *ex vivo* perfusion. The modified strategy was proposed to reduce normothermic *ex vivo* perfusion period, provide 30–45 min of organ stabilizing period before implanting, and reduce the second warm ischemic time, as well as secure the additional reperfusion time. It is anticipated to reduce the cardiopulmonary bypass time and aortic cross clamp time by optimizing the timing of the initiation of cardiopulmonary bypass and aortic cross clamp together with the timing of turning off *ex vivo* perfusion devices.

We proposed a modified strategy, as shown in Figure 1, aiming to reduce the normothermic *ex vivo* perfusion period, provide 30–45 min of the organ-stabilizing period before implantation, reduce the second warm ischemic time, and secure additional reperfusion time (10). This method can reduce the cardiopulmonary bypass and aortic cross-clamp times by optimizing the timing of the initiation of cardiopulmonary bypass and aortic cross-clamp, together with the timing of turning off the *ex vivo* perfusion device (10). We anticipate that the overall outcomes will improve transplantation methods with reduced cardiopulmonary bypass and aortic cross-clamp times.

Owing to the shortage of donor hearts, this case illustrates that expanding the donor acceptance criteria to include more distant donor locations and enrolling recipients with extended criteria is feasible. The expansion of heart transplantation from DCDs would maximize transplantation opportunities and reduce the time spent on transplantation waiting lists. Considering the increasing number of patients with end-stage heart failure awaiting cardiac transplantation, we believe that a regulated normothermic *ex vivo* perfusion device for DCDs is a useful strategy for maximizing organ allocation in select recipients.

## Author’s note

This subject was enrolled in the OCS Heart Perfusion Post-Approval Registry (NCT 05047068). The OCS Heart system is FDA approved for commercial use and patients will be followed per transplant center's standard of care protocols. A waiver of consent has been granted for the data collection by WCG IRB.

## Data Availability

The original contributions presented in the study are included in the article/Supplementary Material, further inquiries can be directed to the corresponding author.
